# A single-blind, parallel-group randomised trial of a Technology-assisted and remotely delivered Cognitive Behavioural Therapy intervention (Tech-CBT) versus usual care to reduce anxiety in people with mild cognitive impairment and dementia: study protocol for a randomised trial

**DOI:** 10.1186/s13063-023-07381-2

**Published:** 2023-06-20

**Authors:** Nadeeka Dissanayaka, Deborah Brooks, Peter Worthy, Leander Mitchell, Nancy A. Pachana, Gerard Byrne, Syed Afroz Keramat, Tracy Comans, Sally Bennett, Jacki Liddle, Mark D. Chatfield, Annette Broome, Joanne Oram, Kanaganayagam Appadurai, Elizabeth Beattie, Tiffany Au, Teagan King, Kimberley Welsh, Ann Pietsch

**Affiliations:** 1 UQ Centre for Clinical Research, Faculty of Medicine, The University of Queensland, Brisbane, Australia; 2 School of Psychology, Faculty of Health and Behavioural Sciences, The University of Queensland, Brisbane, Australia; 3 Metro North Hospital and Health Services, Brisbane, Australia; 4 Centre for Health services Research, Faculty of Medicine, The University of Queensland, Brisbane, Australia; 5 School of Rehabilitation and Behavioural Science, Faculty of Health and Behavioural Sciences, The University of Queensland, Brisbane, Australia; 6 Metro South Hospital and Health Services, Brisbane, Australia; 7 School of Nursing, Faculty of Health, Queensland University of Technology, Brisbane, Australia; 8 Consumer and Community Involvement Group, UQ Centre for Clinical Research, Faculty of Medicine, The University of Queensland, Brisbane, Australia

**Keywords:** Dementia, Mild cognitive impairment, Anxiety, Psychotherapy, Cognitive behavioural therapy, Telehealth, Technology, Digital voice assistant, Randomised controlled trial

## Abstract

**Background:**

Anxiety is commonly experienced by people living with mild cognitive impairment (MCI) and dementia. Whilst there is strong evidence for late-life anxiety treatment using cognitive behavioural therapy (CBT) and delivery via telehealth, there is little evidence for the remote delivery of psychological treatment for anxiety in people living with MCI and dementia. This paper reports the protocol for the Tech-CBT study which aims to investigate the efficacy, cost-effectiveness, usability and acceptability of a *tech*nology-assisted and remotely delivered *CBT* intervention to enhance delivery of anxiety treatment for people living with MCI and dementia of any aetiology.

**Methods:**

A hybrid II single-blind, parallel-group randomised trial of a Tech-CBT intervention (*n* = 35) versus usual care (*n* = 35), with in-built mixed methods process and economic evaluations to inform future scale-up and implementation into clinical practice. The intervention (i) consists of six weekly sessions delivered by postgraduate psychology trainees via telehealth video-conferencing, (ii) incorporates voice assistant app technology for home-based practice, and (iii) utilises a purpose-built digital platform, My Anxiety Care. The primary outcome is change in anxiety as measured by the Rating Anxiety in Dementia scale. Secondary outcomes include change in quality of life and depression, and outcomes for carers. The process evaluation will be guided by evaluation frameworks. Qualitative interviews will be conducted with a purposive sample of participants (*n* = 10) and carers (*n* = 10), to evaluate acceptability and feasibility, as well as factors influencing participation and adherence. Interviews will also be conducted with therapists (*n* = 18) and wider stakeholders (*n* = 18), to explore contextual factors and barriers/facilitators to future implementation and scalability. A cost-utility analysis will be undertaken to determine the cost-effectiveness of Tech-CBT compared to usual care.

**Discussion:**

This is the first trial to evaluate a novel technology-assisted CBT intervention to reduce anxiety in people living with MCI and dementia. Other potential benefits include improved quality of life for people with cognitive impairment and their care partners, improved access to psychological treatment regardless of geographical location, and upskilling of the psychological workforce in anxiety treatment for people living with MCI and dementia.

**Trial registration:**

This trial has been prospectively registered with ClinicalTrials.gov: NCT05528302 [September 2, 2022].

## Administrative information

**Table:** 

Title {1}	A single-blind, parallel-group randomised trial of a Technology-assisted and remotely delivered Cognitive Behavioural Therapy intervention (Tech-CBT) versus usual care to reduce anxiety in people with mild cognitive impairment and dementia: study protocol for a randomised trial
Trial registration {2a and 2b}	ClinicalTrials.gov registry number: NCT05528302
Protocol version {3}	Protocol version 4.0 (January 2023)
Funding {4}	Australian Government MRFF Dementia Aging and Aged Care Mission Grant 2020
Author details {5a}	^1^ UQ Centre for Clinical Research, Faculty of Medicine, The University of Queensland, Australia ^2^ School of Psychology, The University of Queensland, Australia ^3^ Centre for Health Services Research, Faculty of Medicine, The University of Queensland, Australia ^4^ School of Rehabilitation and Behavioural Sciences, The University of Queensland, Australia ^5^ Metro South Hospital and Health Services, Australia ^6^ Metro North Hospital and Health Services, Australia ^7^ School of Nursing, Queensland University of Technology ^8^ Consumer and Community Involvement Group, Centre for Clinical Research, The University of Queensland, Australia
Name and contact information for the trial sponsor {5b}	Dr Jodi Clyde-Smith, Executive Director of Research, The University of Queensland
Role of sponsor {5c}	The sponsor and funders played no part in study design; and will play no part in the collection, management, analysis, and interpretation of data; writing of the report; and the decision to submit the report for publication

## Introduction

### Background and rationale {6a}

Dementia is an overarching term used to label neurocognitive disorders of various aetiological subtypes, including Alzheimer’s disease, vascular dementia, dementia with Lewy bodies, dementia due to Parkinson’s disease, frontotemporal dementia, and dementia due to traumatic brain injury [1]. Mild cognitive impairment (MCI) is considered to be a prodromal phase of dementia [1]. In either mild or major dementias, cognitive domains are significantly impacted which negatively affect an individual’s ability to live independently and care for themselves. Consequently, there are many social, emotional and economic implications for people diagnosed with MCI or dementia as well as their care partners [2].

Anxiety is common in people living with MCI and dementia, with a prevalence of up to 38% [3, 5]. Symptoms of anxiety can be observed in the prodromal phase, at the time of diagnosis, as well as in later stages of dementia [6]. Some behavioural symptoms accompanying dementia, such as wandering or agitation, might signify anxiety [7]. Impacts of anxiety include accelerated cognitive decline [8, 10], increased aggressive behaviours [11], and an increased risk of suicide [12, 13]. Anxiety may also negatively influence the quality of life (QoL) of the individual with cognitive impairment and their care partners [14], increase the risk of early institutionalisation [15, 16], and amplify economic burden [17]. Despite this, anxiety is often given little attention in the context of other behavioural and psychological symptoms in people with cognitive impairment.

Cognitive Behavioural Therapy (CBT) has been traditionally considered gold standard in managing anxiety in the general population, with growing evidence for its efficacy in people living with dementia [18, 21]. To date, there has only been one well-reported protocol for a clinical trial treating anxiety in people living with dementia using CBT [22], however, positive treatment effects decreased from 3 to 6 months follow-up. Long-term maintenance of outcomes from psychotherapy for anxiety can thus be problematic in people living with MCI and dementia, without incorporating ongoing practice into the person’s everyday routine. Furthermore, psychological interventions to treat anxiety in MCI and dementia can be complicated by progressive neurodegenerative processes. Developing a psychological intervention that reduces anxiety symptoms and can be broadly used throughout the duration of the disease may contribute to better overall management.

Delivery of interventions to people living with MCI and dementia and their care partners via telehealth (e.g. telephone or video-conferencing) and technology (e.g. specifically designed platforms and apps accessed via computers, tablets, and smartphones) have started to gain traction due to their potential reach and accessibility [23, 25]. Attending in-person psychotherapy sessions can be prohibitive and exhausting, especially for those living in regional, rural or remote areas who do not have access to nearby services and therefore must experience increased travel time and costs [23, 24]. In the last few years, telehealth delivery rapidly increased out of necessity during COVID-19 lockdowns and a reduction in in-person support from health and social services [26]. Lack of services and increased social isolation for people living with MCI and dementia during the COVID-19 pandemic has increased feelings of anxiety and depression [26]. It is therefore important to establish new practices which would facilitate psychotherapeutic contact hours without the necessity of in-person sessions. Psychotherapy conducted via telehealth video-conferencing, supported with other technology, might assist people with getting the help they need to cope with their anxiety. Remotely delivered psychotherapy interventions have shown good efficacy in treating anxiety and depression [27]. Many technology-based interventions have incorporated components of psycho-education, cognitive behavioural therapy and skills training, with potentially small but significant effects on depression for people living with dementia and their carers [24, 25]. Evidence for the telehealth delivery of such interventions and their impact on anxiety in people living with MCI and dementia is still scare, however [26]. Further research is required to establish the efficacy of remotely delivered psychotherapy in this population. Additional factors such as access to stable and high-speed internet services; cost of technology devices; self-efficacy, acceptability and usability of technology by people living with MCI and dementia as well as the physical barrier that a screen inevitably creates between a therapist and their client must also be considered.

This paper reports the protocol for the Tech-CBT study which aims to investigate the efficacy, cost-effectiveness, usability and acceptability of a *tech*nology-assisted and remotely delivered *CBT* intervention to enhance delivery of anxiety treatment for people living with MCI and dementia. The Tech-CBT study combines a modified CBT approach for treatment of anxiety in older adults with cognitive impairment [28], and treatment of anxiety in older adults with Parkinson’s disease [29]. Due to the progressive characteristic of cognitive impairment, CBT techniques which require substantial cognitive domain capacity, such as Socratic questioning and formulation of underlying automatic thoughts and negative appraisals, are not incorporated in the current intervention. Instead, relaxation and calming techniques will be implemented to equip participants with adaptive coping strategies to be used in a broad spectrum of anxiety-provoking situations. Long-term active coping strategies, such as relaxation, have been previously associated with higher positive affect and better psychosocial functioning in older adults residing in nursing homes [30].

### Objectives {7}

The aims of the study are to:

1. Evaluate the efficacy of the Tech-CBT intervention compared to usual care for people living with MCI and dementia.
2. Evaluate outcomes for care partners (where available).
3. Evaluate the usability and acceptability of a technology platform that supports the delivery of CBT using telehealth as well as access to therapy resources by people living with MCI and dementia in-between therapy sessions.
4. Undertake a process evaluation to assess treatment fidelity, dose and contextual factors that may influence effectiveness, and inform future implementation in community and memory clinic dementia care settings.
5. Conduct a within-trial stochastic cost-utility analysis from the Australian health care system perspective to evaluate the cost-effectiveness of the intervention compared to usual care.

#### Hypotheses

Compared to usual care, the Tech-CBT intervention will significantly reduce anxiety and improve QoL in persons with MCI and dementia (Aim 1), as well as reduce care partner burden and improve care partner QoL (Aim 2). The process evaluation will demonstrate that person-specific (e.g. level of cognitive impairment), carer-specific (e.g. baseline carer burden), and contextual factors (e.g. recruitment source) will influence attendance of Tech-CBT sessions (dose), which will impact outcomes of the RCT (Aims 3 and 4). The Tech-CBT intervention has the potential to become the dominant strategy over usual care for people living with MCI and dementia (Aim 5).

### Trial design {8}

**Fig. 1:**
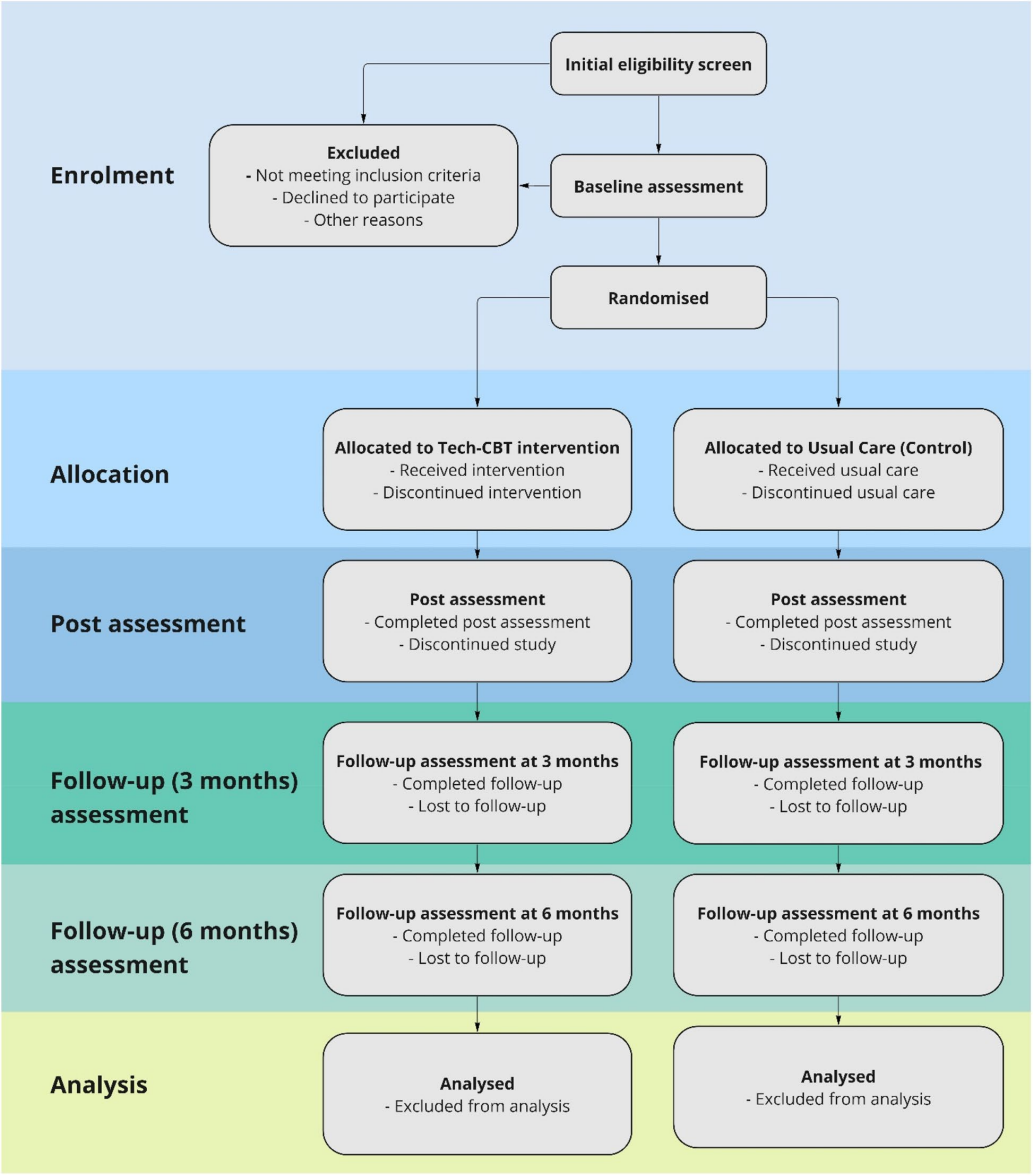
CONSORT flow diagram

This study will use a hybrid II single-blind, parallel-group randomised controlled superiority trial design to test the efficacy of the Tech-CBT intervention versus usual care, with concurrent economic evaluation and an inbuilt mixed-methods process evaluation. A CONSORT-style flow chart for the trial is shown in Fig. 1.

## Methods: participants, interventions and outcomes

### Study setting {9}

The study will recruit people living with MCI and dementia and anxiety from the community, public and private hospital outpatient clinics and memory clinics across Australia. Please see the clinical trials registry for the latest information on recruitment sites: https://www.clinicaltrials.gov/ct2/show/NCT05528302.

### Eligibility criteria {10}

Inclusion criteria are:

1. Persons aged 18 years or over able to communicate in English.
2. Persons with a diagnosis of mild cognitive impairment (MCI) or dementia of any aetiology based on a previous diagnosis by a clinician or scoring above threshold (≤ 32; MCI ≤ 32 and dementia ≤ 27) for cognitive impairment in the Modified Telephone Interview for Cognitive Impairment (TICS-M) [31].
3. Persons who report subjective complaints of anxiety and/or screens positive for anxiety on either or all of the following measures: Geriatric Anxiety Inventory (GAI) [32] and Rating Anxiety in Dementia Scale (RAID) [33].
4. Their care partners, when available. [While the participation of care partners is desirable, participants who do not have a care partner or whose care partners do not wish to participate in the study, will be included should they have the capacity to perform the activities included in the therapy].

Exclusion criteria include:

1. Persons with severe dementia.
2. Persons unable to communicate or complete questionnaires.
3. Persons who have a risk of suicide as identified by the University of Queensland Suicidal Risk Assessment administered at the UQ Psychology clinic which has been modified for research use.
4. Persons with comorbid psychiatric conditions.

People with a comorbid diagnosis of depression will be included in the study, provided that their primary diagnosis or presenting symptom is anxiety. Should depression be identified as the main presenting issue and anxiety intervention reported as ineffective, a referral to a psychologist will be provided and the person will be excluded from the study. People on existing anxiolytics or antidepressants will be allowed to participate. Participants will be instructed not to pursue concurrent psychological therapy over the trial duration and a trial commencement notification will be sent to their GP / referring clinician to increase compliance. Changes to medication will be checked at each therapy and assessment session. If a participant commences psychotherapy during the trial period, they will be withdrawn from the study. Participants’ prior history of depression/anxiety treatment, and any other relevant psychiatric diagnoses, will be recorded.

Study entry will be determined by discussions with the core investigator team, consisting of clinicians and experienced researchers.

### Who will take informed consent? {26a}

Written or online informed consent will be obtained from people living with MCI or dementia and care partners; alternately in instances where the person does not have the capacity to provide informed consent, proxy consent by their legal representatives will provide consent on behalf of the individual, following acceptable methods [34]. A researcher will read through the information sheet with each potential participant and their care partner, guardian and/or authorised person by law. The researcher will then administer the Evaluation to Sign Consent Measure, a reliable and valid measure to evaluate the capacity to consent to participate in research [35]. If the individual is able to demonstrate an understanding of the research study and the impact that participation will have on her or him, then capacity is assumed and consent to participate can proceed with the participant. Participation in the study will be voluntary. Assent will be obtained at the beginning of each therapy session and/or assessment. Participants can withdraw from the study at any time without affecting the quality of future care, or relations with the members of the University, hospitals they are attending or organisations or networks they are involved with.

### Additional consent provisions for collection and use of participant data and biological specimens {26b}

 On the consent form, participants will be asked if they agree to use of their data should they choose to withdraw from the trial. Participants will also be asked for permission for the research team to share relevant data with people from the Universities taking part in the research or from regulatory authorities, where relevant. This trial does not involve collecting biological specimens for storage.

## Interventions

### Explanation for the choice of comparators {6b}

The comparator for this study is usual care, which often translates to no treatment for anxiety, as anxiety is not routinely recognised or treated in people living with MCI and dementia in community or memory clinics in Australia.

### Intervention description {11a}

**Fig. 2:**
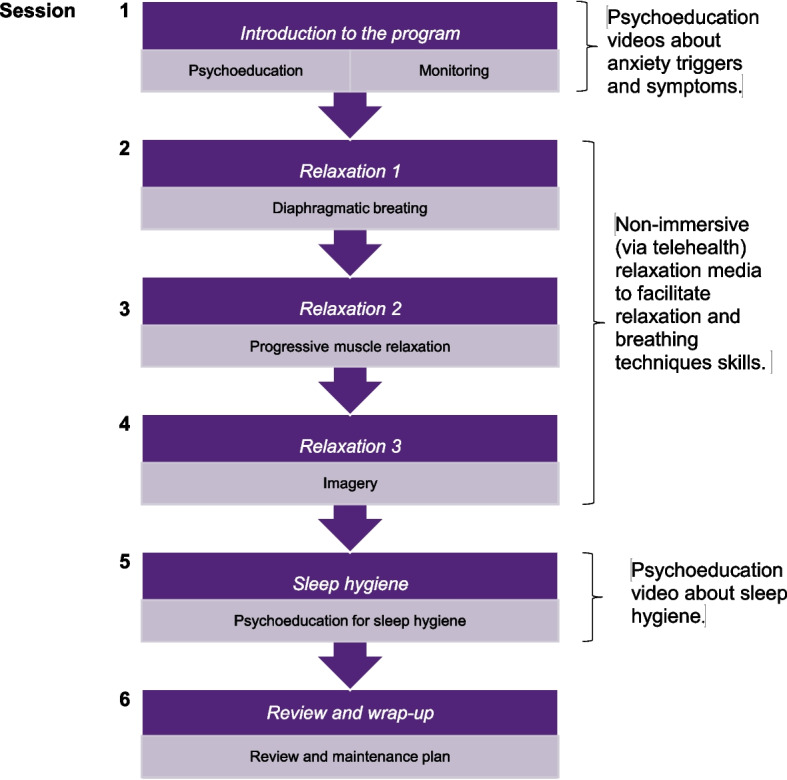
Tech-CBT session outline

Tech-CBT consists of six weekly psychotherapy sessions (60–90 min each) which include psychoeducation, relaxation techniques, sleep hygiene strategies and videos of naturalistic scenes (e.g. walking in a relaxing place). The videos were co-designed with our Consumer and Community Involvement Group (CCIG) representatives. The Tech-CBT intervention is illustrated in Fig. 2.

The techniques previously formulated in a CBT therapist manual [28] will be facilitated through technology that aims to make access to therapy sessions and self-managed practice more accessible. Providing a single platform that is deployable as a number of ‘applications’ (smart assistant and web applications) that enables participants to participate in therapy sessions with their therapist using video-conferencing, access therapy media, record reflections and experiences, and communicate with their therapist may reduce both the practical burden of using multiple applications and manual processes and the consequent cognitive load. Allowing participants to choose a preferred device (smartphone, tablet, desktop computer, smart assistant) for interaction with the program may support continued practice by meeting the individual needs and preferences of participants. The use of voice as an interaction mechanism aims to support participant immersion in the therapy reducing the need to shift focus away from the therapy as is the case with other interaction mechanisms. Care partners (when available) will be instructed to actively engage in therapy sessions as well as in between-session home tasks to support the person with cognitive impairment [36]. The security incorporated into the Tech-CBT platform aims to ensure that the participants’ privacy and confidentiality is strictly protected at all times. Tech-CBT will be accessible to participants via (a) Samsung tablets or (b) Amazon Echo Smart Assistants mailed to participants in the intervention group. All of these devices will be connected to individual service provider accounts (Amazon) that will be managed and paid for by the study. Mobile 4G WiFi Hotspots equipped with pre-paid SIM plans will also be provided. In this way, people who do not have financial resources available to them at the time of the study will not be excluded. Participants will receive technical support during the trial period using a dedicated telephone hotline.

The intervention will be delivered by postgraduate trainees completing advanced psychology training at The University of Queensland (UQ) Australia, who at a minimum are provisionally registered psychologists with the Psychology Board of Australia (Australian Health Practitioner Regulation Agency) or registered mental health counsellors or counselling psychotherapists undergoing advanced postgraduate training at UQ. All trainees will be supervised by registered psychologists at UQ. Therapists will undergo specific training focused on treatment of anxiety in persons with cognitive impairment and telehealth delivery using a digitalised training package. A pre-post design will be used to determine the effect of the training package on psychology trainees’ knowledge and confidence in using Tech-CBT in people living with MCI and dementia and for telehealth delivery of the intervention, and the acceptability of training ascertained.

#### Control group

Participants allocated to the control group will receive usual care and a check-in email, phone call or video call (depending on preference) from a researcher approximately after 3 weeks of completing the initial questionnaires to check whether there are any changes to their usual care and remind them of the post-assessment. At the end of the study, participants will be offered a report to their GP from the post-assessment to help create a mental health plan and/or a list of psychologists by Australian state, based on their preference.

### Criteria for discontinuing or modifying allocated interventions {11b}

There will be no special criteria for discontinuing or modifying allocated interventions.

### Strategies to improve adherence to interventions {11c}

Both intervention and control groups will receive a check-in by the research team (via email, phone or video-call) approximately after 3 weeks of completing the initial questionnaires to see whether there are any changes to their usual care (control) or difficulties using the technology provided (intervention). Therapists will send reminders to participants before each session. Therapists can view participant interactions in-between sessions, such as anxiety monitoring logs, to discuss in subsequent sessions.

### Relevant concomitant care permitted or prohibited during the trial {11d}

Participants will be instructed not to pursue concurrent psychological therapy over the trial duration and a trial commencement notification will be sent to their GP/referring clinician to increase compliance. Changes to medication will be checked at each therapy and assessment session. If a participant commences psychotherapy during the trial period, they will be withdrawn from the study.

### Provisions for post-trial care {30}

At the end of the study, participants will be offered a report to their GP from the post-assessment to help create a mental health plan and/or a list of psychologists by Australian state, based on their preference.

### Outcomes {12}

Pre-, post- and 2 × follow-up assessments at 3- and 6-months post-intervention will be administered in two ways: (1) by self-report surveys completed by the person with dementia or care partner, online or via hard-copy and (2) by trained assessors via video-conferencing, using validated instruments and blinded to randomisation group. Data collection timepoints can be seen in Table 1.

**Table 1:** SPIRIT participant timeline for the Tech-CBT study

	Study period							
	EOI	Pre-assessments	Allocation	Intervention	Post-assessments			Close-out
*Time Point*	*T1* *Baseline*		*Week 1*	*Weeks 2–7*	*T2* *Week 8*	*T3* *Week 20*	*T4* *Week 32*	
Recruitment								
**Eligibility screen *(telephone or zoom*):**								
-Modified Telephone Interview for Cognitive Impairment (TICS-M)	X							
-Geriatric Anxiety Inventory (GAI)	X				X	X	X	
Informed consent	X	X						
Allocation			X					
Treatment								
Tech-CBT				X				
Usual care				X				
Participant assessments								
*** (Self-report online or hard copy)*** -Demographics -Diagnosis -Comorbid medical conditions -Medication -Psychotherapy details -Lawton Instrumental Activities of Daily Living (IADL)		X						
***Primary outcome measure (interview):***								
-Rating Anxiety in Dementia (RAID)		X			X	X	X	
***Secondary outcome measures (interview):***								
-Quality of life in Alzheimer’s disease (QoL-AD)		X			X	X	X	
***Secondary outcome measures (self-report online or hard copy):***								
-Penn State Worry Questionnaire Abbreviated (PSWQ-A)		X			X	X	X	
-Perceived Stress Scale (PSS-14)		X			X	X	X	
-Geriatric Depression Scale (GDS-15)		X			X	X	X	
-Modified Resource Utilisation in Dementia Questionnaire (RUD*) if the person does not have a care partner to complete*		X			X	X	X	
***Additional measures for patients with Parkinson’s disease (self-report online):***								
-Parkinson’s Anxiety Scale (PAS)		X			X			
-Parkinson’s Disease Specific Anxiety Inventory (PD-SAI)		X			X			
-Patient Reported Outcomes in Parkinson’s Disease (PRO-PD)		X			X	X	X	
Care partner assessments								
***Measures for care partners (self-report online):***								
-Demographics		X						
-Depression, Anxiety, and Stress Scale (DASS-21)		X			X			
-Zarit Burden Interview (ZBI)		X			X	X	X	
-Quality of Life in Alzheimer’s Disease (QoL-AD carer) Note: QOL-AD carer will only be administered if the participant displays difficulty in completing it themselves		X			X	X	X	
-Assessment of quality of life (AQoL)		X			X	X	X	
-Resource Utilisation in Dementia Questionnaire (RUD)		X			X	X	X	

#### Primary outcome measure

Change in *anxiety* at post intervention compared to baseline as measured by the RAID [33], a validated scale with superior psychometric properties in dementia compared to other anxiety rating scales [37]; used in past anxiety clinical trials [21]. Score ranging between 0 and 54 (lower score indicates better outcomes).

#### Secondary outcome measures

##### Quality of life

1. Change in quality of life from baseline as measured by the quality of life in Alzheimer’s disease (QoL-AD) [38]. Score ranging between 0 and 52 (higher score indicates better outcomes).

##### Components of anxiety

2. Change in anxiety from baseline as measured by the GAI [32]. Score ranging between 0 and 20 (lower score indicates better outcomes).
3. Change in worry from baseline as measured by the abbreviated version of the Penn State Worry Questionnaire (PSWQ-A) [39]. Score ranging between 8 and 40 (lower score indicates better outcomes).
4. Change in stress as measured by the Perceived Stress Scale (PSS-14) (Bradford et al., 2013). Score ranging between 0 and 56 (lower score indicates better outcomes).

##### Depression

5. Change in depressive symptoms as measured by the Geriatric Depression Scale (GDS-15) [40]. Score ranging between 0 and 15 (lower score indicates better outcomes).

##### People living with Parkinson’s disease and MCI or dementia.

6. Change in anxiety from baseline as measured by the Parkinson’s Anxiety Scale (PAS) [41] . Score ranging between 0 and 48 (lower score indicates better outcomes).
7. Change in anxiety from baseline as measured by the Parkinson's disease Specific Anxiety Inventory (PDSAI) [42]. Score ranging between 0 and 40 (lower score indicates better outcomes).
8. Change in Parkinsonism symptomology as measured by the Patient Reported Outcomes in Parkinson’s Disease (PRO-PD) [43]. Score ranging between 0 and 3500 (lower score indicates better outcomes).

##### Care partners

9. Change in carer burden as measured by the Zarit Burden Inventory (ZBI) [44]. Score ranging between 0 and 88 (lower score indicates better outcomes).
10. Change in carer quality of life from baseline as measured by the assessment of quality of life (AQoL-6D)[45]. Score ranging between 20 and 99 (lower score indicates better outcomes).
11. Change in carer depression and anxiety symptoms from baseline as measured by the Depression Anxiety Stress Scales (DASS-21) [46]. Score ranging between 0 and 126 (lower score indicates better outcomes).

### Participant timeline {13}

The SPIRIT participant timeline for the study can be seen in Table 1.

### Sample size {14}

The study aims to have a sample of 48 (24 in each arm) people living with MCI or dementia with complete data at T1 and T2 (primary time point) providing 80% power to detect a 5-point between-group difference in primary outcome changes (RAID) using a t-test assuming (conservative) SD of 6 points based on previous CBT study in dementia using RAID [33], and a two-sided 5% significance level. Anticipating 30% attrition based on maximum attrition rates observed in prior studies, the study aims to enrol 70 people. Care partners will also be included where possible; however, people living with MCI or dementia may participate without a care partner.

### Recruitment {15}

The study will recruit participants from the community (including the Australian StepUp for Dementia Research database), public and private hospital outpatient clinics and memory clinics. All potential participants will be given the information sheet and consent form during the recruitment process. For participating geriatric, neurology and memory clinics, the consulting clinician will decide who they will pass study information on to, based on their assessments and psychological testing. Only those who have been identified as having MCI or mild to moderate dementia and anxiety will be offered the study information pamphlet. The clinician will check that these persons/carers agree to have their contact details sent to the study team before sending them via online form. The research team will then contact those persons/carers to discuss potential recruitment to the trial and answer any questions.

## Assignment of interventions: allocation

### Sequence generation {16a}

Following baseline assessment, eligible participants will be randomised (1:1 ratio) to intervention or usual care. The randomisation list was created by an independent statistician using computer-generated random numbers and blocks of various sizes.

### Concealment mechanism {16b}

The allocation sequence in the Research Electronic Data Capture (REDCap) database is concealed from all study personnel (except the Clinical Research Coordinator), including the investigators, field staff, and participants.

### Implementation {16c}

The clinical research coordinator will enrol participants and assign participants to interventions.

## Assignment of interventions: blinding

### Who will be blinded {17a}

All assessors will be blinded to the intervention/control arm. There can be no blinding of participants, care supporters, therapists or the central trial team.

### Procedure for unblinding if needed {17b}

The design is open-label with only outcome assessors being blinded so unblinding will not occur.

## Data collection and management

### Plans for assessment and collection of outcomes {18a}

People living with MCI or dementia and their care partners will complete baseline assessments. After 6 weeks, all participants will complete post- (T2; primary time point), 3-month (T3) and 6-month (T4) follow-up from post-assessment.

#### Initial screen

The initial screen consists of two parts. The first is a screening questionnaire conducted online or by telephone to check the person’s eligibility based on their experiences with anxiety and current diagnoses. The second is conducted over telephone or video-conference to check the person has a cognitive impairment or dementia. This includes administration of the TICS-M [31] and the GAI [32].

#### Baseline assessment

Participants who complete the initial screen and agree to participate will complete an electronic questionnaire with electronic consent (paper–pencil versions will also be available). This includes demographics, details of diagnosis, any comorbid medical conditions, medications, any psychotherapy details, the PSWQ-A [39], the PSS-14 [47], the GDS-15 [40], the Lawton Instrumental Activities of Daily Living (IADL; [48], and the Modified Resources Utilisation in Dementia Questionnaire (RUD) if the participant does not have a care partner to complete [49]. Participants who have a diagnosis of Parkinson’s disease will also complete the PAS [41], PD-SAI [42] and the PRO-PD [43]. Participants will be asked to obtain assistance from their care partner (if required) or researcher when completing questionnaires, and to take breaks in between assessments to minimise fatigue.

Care partners will complete a self-report questionnaire online that includes demographics, DASS-21 [50], ZBI [51], the AQoL-6D [45] and the RUD [49].

Within two weeks, participants will also complete an assessment interview via video-conferencing. This includes administration of the RAID [33], and the QoL-AD [38].

#### Post-assessments

Both the self-report questionnaires and measures administered by a trained assessor during the clinical interview are part of the post-assessments at timepoints 2 (week 8), 3 (week 20) and 4 (week 32).

#### Process evaluation

The process evaluation will use mixed methods to understand the processes and conditions which may influence program effectiveness as recommended in the Medical Research Council’s Process Evaluation guidance [52], and to help develop a roadmap for future implementation.

##### Quantitative data

Data regarding reach will be collected using trial recruitment records that report numbers invited and recruited from each source, numbers per setting (community, memory clinics) and geographical location. Dose (attendance at each session) and intensity (length of each session) will be recorded by therapists at each session. Quantitative data will be analysed using descriptive statistics (e.g. for reach) and regression models (e.g. to identify factors influencing participation, completion, and outcomes), as appropriate.

##### Qualitative evaluation of the use of technology-assisted psychotherapy

Any practical issues arising in each session (e.g. technological issues or other) and therapist observation of client engagement will be recorded in the therapist workbooks, as will contextual factors influencing attendance of sessions (e.g. health factors, prior experience with technology, type of setting). This qualitative component will use qualitative description methods [53] as it is well suited to mixed methods research [54].

Qualitative interviews will be held with a purposive sample of participants. These semi-structured interviews will seek overall feedback on the use and accessibility of the intervention to identify, understand and address factors influencing the therapy delivery for future optimisation of the program. Participants will be purposively sampled for maximum variation from community and hospital clinics. Interviews will be audio-taped and transcribed. Rigour will be enhanced by member checking (sending a summary discussion to participants for checking/feedback), triangulation, and reflexivity.

Participants with MCI and dementia and their care partners (*N* = 10 dyads of people living with MCI or dementia and their care partners, and 10 people living with MCI or dementia who do not have a care partner in the study) will be interviewed upon completion of the 6-week therapy program and in week 9 onwards. Their experience of the intervention and perceptions of its acceptability and feasibility, and factors influencing participation and adherence (e.g. prior technology experiences, beliefs about the intervention’s benefit, competing priorities, practice of techniques) will be evaluated. This will include questions relating to the Unified Theory of Acceptance and Use of Technology questionnaire (UTAUT-2; [55]. Participants from the control group will also be invited to interview to ascertain their experiences of usual care. Data will be coded and analysed using a data-driven inductive thematic analysis approach [56].

Therapists (*N* = 18) and their supervisors (*N* = 4) will be interviewed to understand their perceptions of the acceptability of the intervention, reasons for any adaptations required, and any unintended consequences. Interviews will draw on the seven domains of the Theoretical Framework of Acceptability [57] (affective attitude, burden, perceived effectiveness, ethicality, intervention coherence, opportunity costs, and self-efficacy) and the Theoretical Domains Framework [58] to explore factors influencing the delivery of Tech-CBT, and to understand barriers and enablers to further scale-up and implementation.

Representatives from our research partners and wider stakeholders (*N* = 16) will be interviewed to explore perceptions of the intervention, contextual factors influencing its use, and barriers/facilitators to future implementation and scalability, informed by the NASSS (non-adoption, abandonment, scale-up, spread, sustainability) framework [59]. The data will be analysed using the framework approach [60], while staying open to themes identified beyond the NASSS framework.

##### Training package evaluation

The acceptability of the therapist training package will be evaluated using the Theoretical Framework of Acceptability (TFA; [57]. The TFA was developed to evaluate the acceptability of healthcare interventions and has been used to assess training programs (e.g. [61, 62]. A mixed-methods approach will be used, combining a semi-structured interview and qualitative questionnaire at three time points: pre-training, post-training, and post-intervention delivery. A framework analysis approach, employing both deductive (based on TFA domains) and inductive processes will be used to analyse the qualitative data [60].

#### Economic evaluation

A cost-utility analysis and associated sensitivity analyses of the Tech-CBT intervention will be undertaken with the primary analysis from the perspective of the Australian public healthcare system. This perspective is the most commonly used economic evaluation method in Australia for policy makers [63]. A secondary analysis will be from a societal perspective since governments are often interested in the costs incurred by society. In this perspective, all costs incurred or saved by the treatment are included, regardless of who experiences them. This perspective includes care partner costs, patient time waiting and receiving care, transport costs, and lost wages resulting from participating in the intervention.

Bootstrapped incremental cost-effectiveness ratios will be calculated to explain the incremental costs to gain one additional unit of benefit (QALY) of using Tech-CBT over usual care. The formula for calculating ICER is as follows.

$$\textbf{ICER}=\frac{{\textbf{C}}_\text{int}-{\textbf{C}}_{\mathbf u\mathbf s\mathbf u\mathbf a\mathbf l}}{{\textbf{QALY}}_\text{int}-{\textbf{QALY}}_{\mathbf{usual}}}=\frac{\Delta\textbf{C}}{\Delta\textbf{QALY}}$$

where, ${\text{C}}_{\text{int}}$ and ${\text{C}}_{\mathrm{usual}}$ represent the cost of the intervention (Tech-CBT), and usual care, respectively. ${\text{QALY}}_{\text{int}}$ and ${\text{QALY}}_{\text{int}}$ refer to the quality-adjusted life years gained from the Tech-CBT intervention, and usual care, respectively.

Cost-utility will be modelled using techniques appropriate for trial data, such as a generalised estimating equation (GEE) with robust standard errors which can adjust for the clustering and non-normal nature of the data [64]. A final parsimonious model will be selected following data examination and measures of model fit. To capture the uncertainty in model parameter estimates, one-way and probabilistic sensitivity analyses (PSA) will be conducted to explore variability in the sampling and population. The results of the PSA will be presented through the cost-effectiveness acceptability curve (CEAC). The CEAC analysis will show the probability that Tech-CBT is cost-effective at varying willingness-to-pay thresholds against per QALY gained.

This study will estimate the intervention costs following bottom-up costing (micro-costing) approach. This approach quantifies the cost of each input consumed in preventing or treating disease. Intervention costs (resources needed for the delivery of the Tech-CBT), primary care (medications, tests, GP, and specialist consultations), secondary care (hospital admissions), and patient costs (travel, lost work time through participating in the interventions, patient recovery time, lost productivity, and informal care) will be calculated for the evaluation purpose. Unit costs of care will be derived from appropriate sources for the care. For primary care, the Medicare schedule with out of pocket will be applied, and average Australian Refined Diagnosis Related Groups applied for hospital stays. Other costs will be applied at appropriate wage standards for the care. For example, unit costs for informal care will be calculated using estimated unpaid work rates, and time lost due to the intervention will measured through average earnings. Costs will be expressed in Australian dollars at 2025 prices, and the outcome of health benefits will be measured through gained QALYs. Utility values will be estimated from the quality of life in Alzheimer’s disease (QOL-AD) instrument using the Australian algorithm and multiplied using the area under the curve method by the time in the trial to derive the QALYs. Cost-effectiveness results will be presented as ICERs at the threshold of per QALY gained. In the Australian system, recommended cost-effectiveness thresholds for a QALY gain is $64,000 [65]. Therefore, Tech-CBT intervention is considered to be cost-effective if the ICER is lower than the willingness-to-pay threshold of $64,000 per QALY. In this evaluation, 5% (standard rate for Australian economic evaluation) discount rate will be applied as the costs and outcomes will be estimated for a 4-year trial period (June 2021–June 2025).

### Plans to promote participant retention and complete follow-up {18b}

Participants will be given AUS $50 electronic gift cards at completion of baseline assessments and another $50 after completing each of the post-assessments in weeks 8, 20 and 32, regardless of whether they are allocated to the intervention or control arm.

### Data management {19}

Data will be managed according to the University’s Research Data Management Policy and The Australian Code for the Responsible Conduct of Research. All data collected will be identifiable in order to make contact with participants. During recruitment, each participant will be given a unique research identification number. Where possible, correspondences regarding participants will be de-identified in a re-identifiable manner using the unique research identification number.

Participants will complete online questionnaires via REDCap; a password-protected electronic data capture program which only research team members will have access to. Data entered into REDcap will be over a secure web connection with authentication and data logging. The REDCap server is hosted by the University of Queensland's secure computing facilities, who also handle data backup. Participants will complete assessments and interviews with researchers via video-conferencing software approved by the participating institutes. Any hard copy documents with personal identifiers will be stored in locked cabinets within swipe card-restricted offices. All other information regarding participants will be stored in the secured UQ Research Data Manager.

All study-related data will be stored in a durable format alongside study metadata, regularly backed up on secured ITS servers. Data will be accessible only to the collaborators recorded on the study record, and via their institutional usernames and passwords. The study metadata will be maintained and updated accordingly throughout the study. At the end of the study, all study-related data and files will be archived for 15 years to comply with Good Clinical Practice (GCP), after this time the information will be destroyed securely.

### Confidentiality {27}

The data generated as a result of the research project will be managed according to UQ’s Research Data Management Policy. This policy was developed to ensure that research data is properly managed according to recommendations made in The Australian Code for the Responsible Conduct of Research and applicable legislation.

Data will be de-identified at recruitment in a re-identifiable manner, with a unique study ID being assigned to each participant. Where possible, the unique study ID will be used in correspondences regarding participants.

All hard-copy documents with personal identifiers will be stored in locked cabinets within swipe card-restricted offices. All other information regarding recruitment, participant and study data will be stored in secured, password-protected databases on the UQ Research Data Manager (UQRDM) and REDCap.

REDcap is a password protected electronic data capture program which only research team members will have access to. Data entered into REDcap occurs over a secure web connection with authentication and data logging. The REDCap system is maintained by the Queensland Clinical Trials and Biostatistics Centre at the School of Population Health, University of Queensland. The REDCap server is hosted by the Faculty of Sciences, University of Queensland's secure computing facilities, who also handle data backup and system redundancy. The web interface is secured by the secure sockets layer protocol.

Project metadata such as the project name and the collaborators who will have access to the data, will be recorded in a project record within UQRDM. All project-related data will be stored in a durable format alongside this project metadata, which will be regularly backed up on secured ITS servers. Data will be accessible only to the collaborators recorded on the project record and will be only accessible via their institutional usernames and passwords. The project metadata will be maintained and updated accordingly throughout the project.

At the end of the study, all study-related data and files will be archived for 15 years to comply with Good Clinical Practice (GCP), after this time the information will be destroyed securely.

### Plans for collection, laboratory evaluation and storage of biological specimens for genetic or molecular analysis in this trial/future use {33}

Not applicable for this study. This trial does not involve collecting biological specimens for storage.

## Statistical methods

### Statistical methods for primary and secondary outcomes {20a}

We will use longitudinal ANCOVA (mixed) models for each outcome variable to estimate between-randomisation-group differences (intervention vs usual care) at T2, T3 and T4, adjusting for the outcome at T1.

### Interim analyses {21b}

There are no interim analyses planned.

### Methods for additional analyses (e.g. subgroup analyses) {20b}

There are no subgroup analyses planned.

### Methods in analysis to handle protocol non-adherence and any statistical methods to handle missing data {20c}

Missing data will not be imputed. All available data will be used in the models.

### Plans to give access to the full protocol, participant-level data and statistical code {31c}

De-identified datasets analysed in this study will be available from the corresponding author on reasonable request and with data transfer formal agreements for further research. Consent to share de-identified information for future research is specified within the participant consent forms. The full study protocol and the statistical code will also be available for future collaborative research.

## Oversight and monitoring

### Composition of the coordinating centre and trial steering committee {5d}

The team responsible for trial set-up, administration, recruitment and day-to-day running of the trial, meets weekly. This team consists of the Clinical Research Coordinator, Principal Investigator, Project Manager, Technology Development Research Fellow, staff responsible for recruitment activities and pre-trial assessments (non-blinded). The trial steering committee consists of all Chief and Associate Investigators and study partners and meets every 6 months.

### Composition of the data monitoring committee, its role and reporting structure {21a}

This is a low-risk intervention and the Human Research Ethics Committee does not require a Data Monitoring Committee.

### Adverse event reporting and harms {22}

#### Risk associated with completing assessments

The nature of the assessment battery, the purpose of performing the tests and participants’ ability to withdraw at any stage will be clearly communicated.

#### Discomforts associated with the study

Participants will be asked to take a break or postpone the assessment to another day within the time frame. Participants can avoid answering questions that they feel uncomfortable to answer.

#### Individual outcomes

Outcomes of the assessments at pre- and post- can be discussed with a clinician. Individual information will be disclosed to participant’s referring clinical specialist, such as geriatrician, neurologist or psychiatrist (if not listed in the study) and/or other nominated physician such as GP under the following circumstances:

1. If any abnormalities are detected (e.g. risk of suicide)
2. If participants wish to discuss their individual results.

Written or electronic informed consent will be gained from participants for this disclosure of information to a third party. In other circumstances (e.g. case reports or presentations) privacy and confidentiality will be protected by de-identifying the participant.

#### Adverse event monitoring and reporting

The study investigator is responsible for recording all adverse events, regardless of their relationship to study intervention. Conditions that are present at screening will not be considered adverse events. All assessors and therapists will complete risk assessment documentation and follow the adverse events reporting procedure established for this project. This includes an immediate report of all Significant Safety Issues (SSI), Suspected Unexpected Serious Adverse Events (SUSARs) and Serious Adverse Events (SAEs) to the trial sponsor and required HREC and Governance offices.

Individual information will be disclosed to participant’s referring clinical specialist, such as geriatrician, neurologist or psychiatrist (if not listed in the study) and/or other nominated physician such as GP.

### Frequency and plans for auditing trial conduct {23}

Audit will be performed by the clinical trial coordinator when recruitment target hits 5, 10, 20, 30, 40 and 50. Generated reports to include recruitment table (e.g. contacted, withdrawals, agreed, and disagreed), study protocol violations, serious adverse events (if any), informed consent of trial participants.

### Plans for communicating important protocol amendments to relevant parties (e.g. trial participants, ethical committees) {25}

All protocol changes are approved by the Metro North Health and University of Queensland Human Research Ethics Committees. Updates are made to the trial registry when required.

### Dissemination plans {31a}

A publication and dissemination plan will be developed to include conference presentations and peer-reviewed journal publications, as well as plain English lay summaries developed with the Consumer and Community Involvement Group. All participants will receive a summary of the trial results.

## Discussion

Whilst anxiety has been identified as an important issue affecting people living with MCI and dementia, there are very few studies evaluating the efficacy and cost-effectiveness of psychotherapy treatments in this population. Furthermore, delivery of psychotherapy via telehealth, and utilising technology such as smartphone apps and smart assistants, has tended to focus on interventions for care partners rather than people living with MCI or dementia themselves. To our knowledge, the Tech-CBT study is the first to address these issues and also aims to provide insights into the usefulness, acceptability, and usability of telehealth and technology to deliver psychotherapy from the perspectives of people living with MCI or dementia, their care partners, and therapists. Further, the process evaluation will inform an implementation plan for translation in the community setting as well as for those attending memory, geriatric, and neurology clinics, in order to enhance the delivery of anxiety treatment with a broad reach for persons with cognitive impairment following the COVID-19 crisis. The implementation plan will translate the findings from the process evaluation into concise, proactive, operable guidelines. It will identify barriers and enablers to the effective use of the platform and provide guidelines for a wide range of identified areas including contextual issues, technology use, therapy delivery, participant engagement, clinical rollout, and sustainability.

## Trial status

Protocol version 4.0 (January 2023). Community recruitment commenced February 2023. Hospital recruitment commenced March 2023. Anticipated completion of recruitment is January 2025.

## Data Availability

PI-Dissanayaka will be the data custodian. Any data required to support the protocol can be supplied upon reasonable request.

## Abbreviations

ANCOVA: Analysis of covariance
AQOL: Assessment of quality of life
CBT: Cognitive behavioural therapy
CCIG: Consumer and Community Involvement Group
CEAC: Cost-effectiveness acceptability curve
DASS: Depression, Anxiety, and Stress Scale
GAI: Geriatric Anxiety Inventory
GCP: Good Clinical Practice
GDS: Geriatric Depression Scale
GEE: Generalised estimating equations
HREC: Human Research Ethics Committee
MCI: Mild cognitive impairment
NASSS: Non-adoption, Abandonment, Scale-up, Spread, Sustainability framework
PAS: Parkinson’s Anxiety Scale
PDSAI: Parkinson’s disease Specific Anxiety Inventory
PRO-PD: Patient Reported Outcomes in Parkinson’s Disease
PSA: Probabilistic sensitivity analyses
PSS: Perceived Stress Scale
PSWQ-A: Penn State Worry Questionnaire
QALY: Quality-adjusted life year
QoL: Quality of life
QoL-AD: Quality of life in Alzheimer’s disease
RAID: Rating Anxiety in Dementia Scale
REDCap: Research Electronic Data Capture database
RUD: Resource Utilisation in Dementia Questionnaire
TICS-M: Modified Telephone Interview for Cognitive Impairment
TFA: Theoretical Framework of Acceptability
UQ: The University of Queensland
UTAUT: Unified Theory of Acceptance and Use of Technology questionnaire
ZBI: Zarit Burden Inventory
